# 

**Published:** 1817-12

**Authors:** 


### CORRESPONDENCE.

In consequence of a pressure of matter in our Original and Review departments, we have been obliged to defer a part of our Selections and Miscellanies, and all the Cases intended for The Porte-Feuille.

A Case of Cynanche Laryngea, requiring Tracheotomy and the continued use of a Canula, with a Drawing, will appear in our next number.

Dr. Sanders's paper on the Relation between the Nervous and Vascular Systems; Mr. Boyd's Report of Diseases on board the Gorgon; Dr. Balfour's Cases of Gout; Mr. M'Carogher's Case of Amputation; and Mr. Smith's paper on Pemphigus. &c. have been received.

Mr. Bailey's Work on Belladonna; and Dr. Thornton's Translation of the London Pharmacopœia, have come to hand.

The Title and Index to this Volume will be given in our next publication.

---

END OF VOL. IV.

### INDEX.

A

ABDOMINAL DISEASE, singular case of 227
Abstinence, prolonged, case of 520
Affusion, cold, useful in convulsive diseases 249
Alibert on dermoid diseases 171
*Alisma plantago* given by the Russians in rabies 144
Alvine concretions, symptoms of 190
———————, diagnosis and treatment of 191
Ambergris, resemblance of an intestinal concretion to 179
Amputation, observations on 314
——— of the penis, case of 339
Anæmia, on the characters and treatment of 244
Aneurism, axillary, case of 78
——— of the aorta communicating with the œsophagus 372
Animal irritation, a cause of scirrhous affection of the stomach 497
Aorta, case of rupture of the 426
Apoplectic cyst, extensive one in the brain 112
Apoplexia hydrocephalica, prevalence of 272
Apothecaries, associated, resolutions of 259
Arden, Dr. on the saline spring at Moira baths 147
Arsenious acid, new method of detecting 349
*Arteria innominata*, case of aneurism of the 164
Arteries, remarks on the ligature of 311
Ascites, case of 240
Azote, on the nutritive properties of substances destitute of 64

B

Baron's, Dr. case of rupture of the brain from hydrocephalus 491
Bell's, Mr. Charles, surgical observations 309
Bladder, case of extroversion of the 239
Bleeding useful in cholera 340
———, extensive, used in pneumonia 460
Blister plaster, new one invented 261
Blood-letting, efficacy of, in disordered action of the heart 89
BOOKS, NEW, ANNOUNCED.—Mr. Bayfield's Treatise on the Art of Cupping, 87. Professor Orfila's Elementary Work on Chemistry, 174. Dr. Bancroft's Sequel to his History of Yellow Fever, 174. Mr. Moore's History of Vaccination, 174. Mr. Whately on Gonorrhœa Virulenta, 174. *Le Regne Animal distribué d'apres son Organization*, 175. *Nosologie Naturelle, par Alibert*, 175. Dr. Johnson's Translation of Mess. *Guilbert and Hallé's* Treatise on the Prolongation of Life, &c. 264. Mr. Williams's Analysis of the Waters at Llandridod, 264. Mr. Bailey on the Use of Belladonna, 264. Dr. Gordon's Outlines of Lectures on Human Physiology 552
BOOKS REVIEWED.—Transactions of the Medical Society of London, 31. Mr. Morrison's Treatise on Tetanus, 43. Dr. Gordon's Observations on the Structure of the Brain, 53, 120. Dr. Spurzheim's Examination of the Objections made in Britain against the Doctrines of Gall and Spurzheim, 53, 117. Mr. Howship's Practical Observations in Surgery and Morbid Anatomy, 108, 218. Dr. Von Embden's Continental Medical Repertory, 135. Mr. Saunders's Anatomy of the Human Ear, 145. Dr. Arden's Treatise on the Saline Springs near Ashby-de-la-Zouch, 146. Dr. Chardel's Monograph on scirrhous Affections of the Stomach, 193, 406. Oracular Communications, addressed to Medical Students, 232. Mr. Good's Physiological System of Nosology, 291, 387. Dr. Burrows's Cursory Remarks on the Mad-House Bill, 301. Mr. Charles Bell's Surgical Observations, 309. Mr. Salt on the Inoculation of Morbid Poisons, 319. Mr. Carpue on the Operation for restoring a lost Nose, 376. Wishart's Translation of Scarpa on Hernia, 395. Mr. Parkinson's Essay on the Shaking Palsy, 401. Dr. Majendie's Elementary Summary of Physiology, 469. Medico-Chirurgical Transactions, vol. 8, Part I. 486. Dr. Johnson on the Influence of the Atmosphere of the British Isles on the Health and Functions of the Human Frame, 515. Dr. Scudamore on the Nature and Cure of Gout 519
Bourne's, Dr. case of disordered action of the heart 89
Boyd's Mr. case of wound of the larynx 357
Brain, observations on the structure of 53
——, large apoplectic cyst found in the 112
——, hydatids found in the ventricles of the 152
——, fatal injury of the, from a slight blow 218
——, affection of the, from suppressed perspiration 219
——, rupture of the, from hydrocephalus 491
Bricheteau's, Dr. case of aneurism 372
Brine bath, description and efficacy of 33
Burrows's, Dr. cursory remarks on the mad-house bill 301

C

Cancer of the womb, case of 136
Cardia, cases of scirrhous affections of the 201
Cerebral affection, cases of 425
*Cerebri hernia*, cases of 487
Charcoal, deleterious properties of the fumes of 193
——— powder used for the cure of phthisis 247
Chardel, Dr. on scirrhous affections of the stomach 198, 496
Chemical irritation, a cause of scirrhous affection of the stomach 497
Children, report of diseases of 266, 450
Cholera cured by bleeding 340
Cholera morbus prevalent among children 455
*Chorea Sancti Viti*, case of 456
*Chrysantheum leucanthemum* used in fluor albus 144
Cinchona, on a preparation of 65
Colon, case of fatal disease of the 154
Concretion, remarkable, found in a tumour 143
————, intestinal, case of 177
————, analysis of 179
Contraction, spasmodic, of the voluntary muscles, case of 193
Convulsion, case of, cured by depletion 114
Convulsive diseases, efficacy of cold affusion in 249
——————, observations on 401
Correspondence, 87, 175, 264, 350, 439, 552
*Cranii basis*, fracture of 21
Cranium, case of fracture of the 353
Cupping glasses employed to relieve diarrhœa 337
*Cynanche tonsillaris*, hints on the treatment of 82

D

Dale, Mr. on the effects of diet in stomach disorders 40
Damant's, Mr. cases of obstruction in the bowels 38
Davis's, Dr. report of diseases of children 265, 450
Deafness, treatment of 34
Death produced by eating eggs 7
Denmark's, Dr. case of *phlegmatia dolens* 1
Dermoid diseases, observations on 171
Diaphragm, cases of rupture of the 326
Diarrhœa relieved by the application of cupping glasses 337
Diet, on the effects of, in stomach disorders 40
Diploma, Edinburgh, copy of the 256
Diseases, remarks on the conversion of 94
Dispensary, universal, report of infantile diseases in the 266, 450
Dobson's, Dr. case of fracture of the cranium 353
*Duparque's*, Dr. case of fibrous substance on the surface of the uterus 76

E

Ear, human, anatomy of the 145
Edinburgh Graduates in 1817, list of 254
Eggs, cases of death caused by eating 7
*Elaterium* useful in gout 37
Emetine, properties of 424
Endemic fever in the Achates, history and treatment of the 435
*Epistaxis*, infallible remedy for 34
Eruption, habitual, driven in upon the brain 218
Erysipelas, prevalence of 452
————, *infantile*, observations on 463
Evacuations, on the utility of in obscure diseases 364
Extroversion of the bladder, case of 239

F

Face, large ossific tumour of 110
——, malformation of the bones of the 111
Felix, Dr. on the conversion of diseases 94
Fergusson's, Dr. inquiry into the origin and nature of yellow fever 492
Fever, West Indian, reports on 10, 14

INDEX.

Fever, yellow, observations on the nature and origin of the 492
Fracture of the *basis cranii*, case of 21
Fumigations, sulphureous, used in cutaneous affections 140
Fungus hæmatodes in a child five years old 459

G

Gangrenous affection of the mouth, cases of 70
Garters a cure for cramps in the legs 35
Glasgow, regulations for medical degrees at 257
Good's, Mr. system of nosology, 291, 387
Gordon's, Dr. observations on the structure of the brain 53
Gout cured by *elaterium* 37
——, mode of successfully treating 166
——, remarks on Dr. Balfour's method of curing 346
——, on the nature and cure of 519
Graduates, Edinburgh, list of 254
Green's, Mr. cases of gout cured by *elaterium* 37
Gunshot wounds, cases of 158

H

Hæmorrhage from extracting a tooth, mode of stopping 81
————, parturient, remarks on 144
———— from the villous coat of the intestines 229
———— from the lower orifice of arteries, cases of 313
Head, case of severe pain in the 113
——, death produced by slight injury of the, after forty years 116
Head ach, periodical, case of 156
————, distressing, case of 252
————, chronic, cured by moxa 334
Heart, observations on the action of the 9
——, case of disordered action of the 89
——, cases of affection of the 222
*Hepar sulphuris*, on the effects of, in croup 140
Hepatic abscess, fatal case of 42
———— affection frequent among children 279
Hernia, treatise on, by Professor Scarpa 395
*Hernia cerebri*, cases of 487
Howship's, Mr. practical observations in surgery 108
———— observations on the morbid structure of bones 492
Hydatids found in the ventricles of the brain 152
———— found in the uterus 363
Hydrencephalus acutus, case of 427
Hydrocele, spontaneous disappearance of 98
Hydrocephalus, chronic, case of 80
Hydrophobia, case of 31
————, with appearances on dissection 36

I & J

Ileum, lacerated, case of 137
Induration and contraction of the stomach, case of 332
Infantile diseases, reports of 266, 450
Influenza, prevalent among children 268
Intestinal affections, observations on 419
————, case of 421
Intestines, hæmorrhage from the villous coats of the 229
Ipecacuanha, experiments on 424
Jackson's, Dr. return of sick of the army in the West Indies 41
Jaws, case of anchylosis of the 109
Johnson, Mr. on a preparation of cinchona 85
————, Dr. on the utility of evacuations and low regimen 364
————, on the influence of the atmosphere 515
Johnstone, Dr. James, biographical notice of 42
*Junonium*, a newly discovered metal 438

K

Kennedy's, Dr. case of inflammation of the psoac muscles 104
————, case of intestinal concretion 177
———— ossiform substance in the vulvo labial texture 465
*Kumis*, on the method of preparing 141

L

Larynx, causes of affection of the 220
————, wound of the, successfully treated 357
Lazzaretto's, Dr. cases of traumatic tetanus 102
Lectures announced.—Dr. Merriman and Dr. Lee, on Midwifery, 87, 263, 552.—At St Thomas's and Guy's Hospitals, 261.—At St. Bartholomew's Hospital, 262.—At St. George's Medical and Chemical School, 262.—At the Middlesex Hospital, 262.—Dr. Adams, on Medicine, 262.—Dr. Clough, on Midwifery, 262.—Dr. Clutterbuck, on Medicine and Chemistry, 263.—Mr. Good, on Nosology and Medicine, 263.—Mr. Taunton, on Anatomy and Surgery, 263.—Mr. Wilson and Mr. Bell, on Anatomy and Surgery, 263.—Mr. Gualtier, on Physiology, 349.—Mr. Guthrie, on Surgery, 349.—Mr.

Pettigrew, on Anatomy, 349.—Dr. Ramsbotham, on Midwifery, 350.—Dr. Uwins, on Medicine, 350.—In the University of Edinburgh, 438.
Ligatures of arteries, remarks on 311
Liver, observations on affections of the 228
Living bodies, classification of 477
Lobstein, Dr. on vaginal hæmorrhages 161
Lungs, case of singular disease of the 224
Lymphatic system, description of a peculiar state of the 498
Lythocele, curious case of 141

M

Mad-house bill, cursory remarks on 301
Majendie's elementary summary of physiology 469
Maxillary cavity, case of dropsy of the 142
Measles, case of, with dissection 99
———, extensive prevalence of 274
Mechanical irritation a cause of scirrhous affection of the stomach 496
Medical miscellanies, 78, 164, 249, 338, 428, 526
Menstrual effusion into the cavity of the uterus 230
Menstruation through the mammæ, case of 143
Mercury, on the use of, in yellow fever 81
Meteorological register at Harwich, 88, 176, 351, 352, 440
Monstrosity, observations on, and species of 408
Moodie's, Dr. case of extrordinary tumour 35
Morbid poisons, on the inoculation of 319
——— structure of bones, observations on 492
Morrison's, Mr. case of measles 99
Morrison's, Mr. cases of death from eating eggs 7
———, Dr. treatise on tetanus 43
Mortimer's, Mr. report on the West India fever 10
Moulson's, Dr. observations on *erysipelas infantile* 463
Mouth, on a gangrenous affection of, peculiar to children 70
Moxa, serviceable in chronic head ach 332
Muscular motion, observations on the laws of 430, 526

N.

Naval and military surgery, remarks upon 428
Nitro-muriatic acid bath, successful employment of 316
Nose, operation for restoring a lost 376
Nosology, new physiological system of 291
———, observations on the study of 441
Nutritive properties of substances destitute of azote 64

O

Obituary 87, 175, 264, 350, 438, 552
Observations, practical, in surgery and morbid anatomy 108
Obstruction, fatal, in the bowels, cases of a dissection 38
Operation for restoring a lost nose 376
Oracular communications 232
Ossiform substance developed in the vulvo-labial texture 465

P

Palmer's, Dr. case of fracture of the *basis cranii* 23
——— case of deleterious effects of charcoal 193
Palsy, shaking, essay on 401
Paracentesis performed 665 times in a woman 143
Paralysis, cases of 416
———, case of 522
Park, Dr. on the laws of muscular motion 430, 526
Parkinson, Mr. on the shaking palsy 401
*Parnassia palustris* useful in strangury 144
Parturient hæmorrhage, remarks on 144
Penis, cauliflower, excrescence of the 338
Petechiæ, appearance of, in hydrencephalus acutus 427
Pharynx, wound of the, successfully treated 357
Philip, Dr. on the action of the heart 9
Philosophical Society of London, anniversary meeting of 86
*Phlegmatia dolens*, case of, in a male 1
*Phthisis psoæ*, fatal case of 139
———, sanguineous, cured by charcoal powder 247
Physic, on the limits between it and surgery 342
Physicians, college of, picture of the Present state of 63
Pneumonia, case of 460
Poisons, morbid, on the inoculation of 319
*Polypodium* employed in hæmorrhage 144

PORTE-FEUILLE, THE—Axillary aneurism, 78.—Chronic Hydrocephalus, 80.

—Replacing extracted teeth in hæmorrhage, 81.—Aneurism of the arteria innominata, 164.—Efficacy of cold affusion in convulsive disorders, 249.—Cicatrization of the vagina, 251. —Distressing head-ache, 252.—Cauliflower excrescence of the penis, with amputation, 338.—Bleeding in severe cholera, 340.—Curious modification of variola by previous vaccine, 341.—Violent cerebral affection, 425.—Rupture of the aorta, 426.—Effects of spirit of turpentine in typhus fever, 427. —Hydrencephalus, in which petechiæ appeared, *ib.*
Psoac muscles, case of acute inflammation of 104
Pyloric scirrhus, leading symptoms of 507
Pylorus, cases of scirrhous affections of the 209

Q

Quarrier's, Dr. report of the wounded on board the Leander 486
Question, prize, of the College of Surgeons 258

R

Rabies canina, fatal case of 283
Regulations for medical degrees at Glasgow 257
Remarks on medical subjects 32
Repertory, the continental medical 135
Report, quarterly, of surgical diseases 309
——— of infantile diseases in the Universal Dispensary 265, 450
Reviewer's Reply to Dr. Von Embden 530
Robertson's, Mr. report on the yellow fever 14
Rolfe's, Mr. case of hydatids in the uterus 363
Roxburgh, Dr. on the use of the *Swietenia febrifuga* 31
Rupture of the brain from hydrocephalus 491
Russians, some of the popular remedies among the 144

S

Salivation, periodical, case of 126
Salt, Mr. on the inoculation of morbid poisons 319
Scarpa's, Antonio, treatise on hernia 395
Scudamore, Dr. on the nature and cure of gout 519
Scirrhous affections of the stomach, progressive stages of 502
Selections 64, 149, 239, 326, 408, 520
Senses, external, disorder of the 522
Serum, peritoneal, analysis of 423
Simpson's, Mr. case of pneumonia 460
Sims's, Dr. remarks on medical subjects 32
Small-pox, on the recurrence of the, after vaccination 258
Spasmodic diseases, observations on 401
*Sponsio academica* at Edinburgh, copy of the 256
Spurzheim, Dr. on the structure of the brain 53
Squire's, Dr. case of impregnation when the vagina was scarcely permeable 35
Stanley's, Mr. cases of *hernia cerebri* 487
Stomach disorders, on the effects of diet in 40
Stomach, cases of scirrhous affections of the 206
———, case of induration and contraction of the 332
———, causes of scirrhous affections of the 496
Stones in the stomach and intestines, cases of 185
Surgeons, college of, prize question of the 258
*Swietenia febrifuga*, on the use of 31

T

Tendon of the patella, rupture of the 422
Tetanus, on the treatment of 44
———, cases of 48
———, traumatic, successfully treated 102
Tooth-ach, infallible cure for the 34
Tracheotomy, unsuccessful performance of 336
Transactions of the London College of Physicians 31
Tumour, extraordinary, case of a 35
Turpentine, spirit of, efficacious as a purgative 427
Typhus contagion may exist between the tropics 494

U

Ure's, Dr. experiments on ambergris 183
Uterus, peculiar fibrous substance on the surface of the 76
———, effusion into the cavity of the 230
———, hydatids found in the 363

V

Vaccination, report of the French Institute on 437
Vagina, case of cicatrization of the 251
Variola modified by previous vaccine 341
Veins, on the circulation of the blood through the 149

Ventricles of the brain, hydatids found in the 152
Vomiting, chronic, cured by mercurial salivation 333
———, spasmodic, remarks on and cases of 508
——— dependent on various causes 512

W

Walshman's, Dr. case of hydrophobia 36
Ward's, Mr. case of fracture of the *basis cranii* 21
Webster's, Mr. case of rabies canina 283
White's, Mr. case of hydrophobia 31
Womb, case of cancer of the 136
Worcestershire Medical Society, resolution of the 87
Wounds of arteries, remarks on 311

Y

Yellow fever, report on, to the Transport Board 14
Yellow fever, on the origin and nature of the 492

Z

Zinc, watery solution of the sulphate of, used in scabies 144

### References to the Plate and Drawing.

Dr. Denmark's Case of Phlegmatia Dolens - - - - - - - 1
Dr. Dobson's Case of Fracture of the Cranium - - - 353

PRINTED BY W. THORNE, RED LION COURT, FLEET STREET.

